# Supplementary material for: Netcombin: An algorithm for constructing optimal phylogenetic network from rooted triplets
Source: PLoS One. 2020 Sep 18;15(9):e0227842. doi: 10.1371/journal.pone.0227842 (PMC7500971; doi:10.1371/journal.pone.0227842)
Supplement: S4 File — (ZIP) [file pone.0227842.s004.zip › SequenceData-Triplet/sequenceData-10-20-30-0-Triplet/length 200, numseq 30, rate 10^-7/10 seq.pdf]

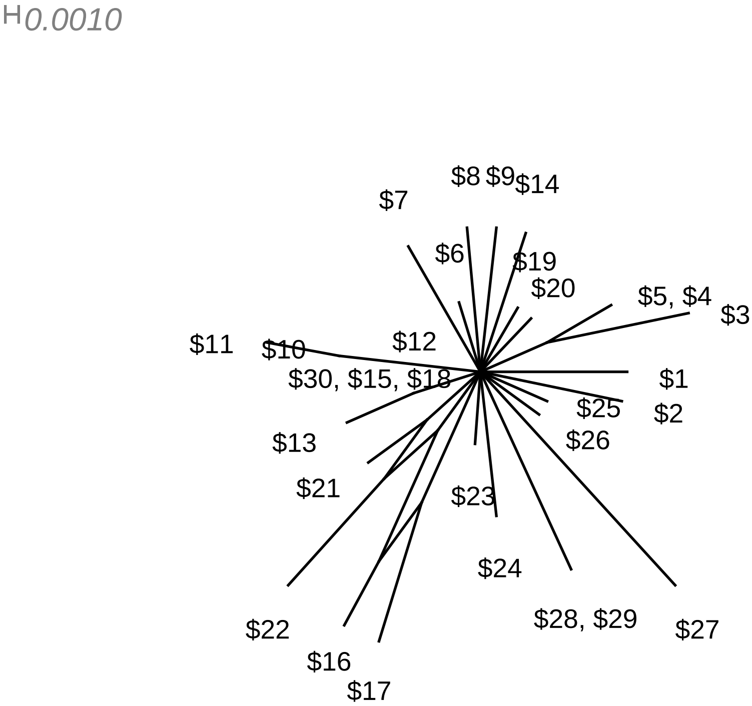

0.0010
$8
$9
$14
$7
$6
$19
$20
$5, $4
$3
$11
$10
$12
$30, $15, $18
$1
$25
$2
$13
$26
$21
$23
$24
$28, $29
$27
$22
$16
$17
